# Tailored interventions to implement recommendations for elderly patients with depression in primary care: a study protocol for a pragmatic cluster randomised controlled trial

**DOI:** 10.1186/1745-6215-15-16

**Published:** 2014-01-09

**Authors:** Eivind Aakhus, Ingeborg Granlund, Jan Odgaard-Jensen, Michel Wensing, Andrew D Oxman, Signe A Flottorp

**Affiliations:** 1 Research Centre for Old Age Psychiatry, Innlandet Hospital Trust, N-2312 Ottestad, Norway; 2 Norwegian Knowledge Centre for the Health Services, Box 7004, St Olavs plass, N-0130 Oslo, Norway; 3 Radboud University Nijmegen Medical Centre, PO Box 9102, 6500 HC Nijmegen, The Netherlands; 4 The Department of Health Management and Health Economics, University of Oslo, PO Box 1130, Blindern, 0318 Oslo, Norway

**Keywords:** Depression, Elderly, Primary care, Tailored interventions, Implementation science, Cluster randomised trial

## Abstract

**Background:**

The prevalence of depression is high and the elderly have an increased risk of developing chronic course. International data suggest that depression in the elderly is under-recognised, the latency before clinicians provide a treatment plan is longer and elderly patients with depression are not offered psychotherapy to the same degree as younger patients. Although recommendations for the treatment of elderly patients with depression exist, health-care professionals adhere to these recommendations to a limited degree only. We conducted a systematic review to identify recommendations for managing depression in the elderly and prioritised six recommendations. We identified and prioritised the determinants of practice related to the implementation of these recommendations in primary care, and subsequently discussed and prioritised interventions to address the identified determinants. The objective of this study is to evaluate the effectiveness of these tailored interventions for the six recommendations for the management of elderly patients with depression in primary care.

**Methods/design:**

We will conduct a pragmatic cluster randomised trial comparing the implementation of the six recommendations using tailored interventions with usual care. We will randomise 80 municipalities into one of two groups: an intervention group, to which we will deliver tailored interventions to implement the six recommendations, and a control group, to which we will not deliver any intervention. We will randomise municipalities rather than patients, individual clinicians or practices, because we will deliver the intervention for the first three recommendations at the municipal level and we want to minimise the risk of contamination across GP practices for the other three recommendations. The primary outcome is the proportion of actions taken by GPs that are consistent with the recommendations.

**Discussion:**

This trial will investigate whether a tailored implementation approach is an effective strategy for improving collaborative care in the municipalities and health-care professionals’ practice towards elderly patients with depression in primary care. The effectiveness evaluation described in this protocol will be accompanied with a process evaluation exploring why and how the interventions were effective or ineffective.

**Trial registration:**

ClinicalTrials.gov: NCT01913236

## Background

The prevalence of depression in the elderly is high [1,2] and increases with age, even within the elderly [3-5]. Elderly patients with depression are to a large extent treated in primary health care, and they prefer to be treated by their general practitioner [6,7]. The risk that an elderly patient with depression develops a chronic episode is estimated to be approximately 30% [8,9]. Depression in the elderly has a negative impact on quality of life, the episodes of the disease are longer, and the risks of hospitalisation and mortality are increased [10]. Medical co-morbidities, which increase with advancing age, have a negative effect on treatment response and prognosis [10,11]. Practitioners’ attitudes towards and experience with depressed elderly patients affect the probability of providing a patient with an adequate treatment strategy [12], and patients’ attitudes and beliefs towards the treatments might affect adherence and outcomes [13,14]. Elderly patients with depression are less likely to be offered a course of psychotherapy [15], and GPs’ latency before reaching a decision with regard to a treatment strategy is longer. In Norway elderly patients are not referred to district psychiatric centres to the same degree as younger adults and when referred, the duration of contact for the treatment is shorter [16]. To our knowledge, psychiatrists and psychologists in private practice treat elderly patients with depression to a very limited degree only. International studies indicate that general practitioners accurately diagnose about 50% of patients with depression [17], and approximately 40% of practice is in accordance with depression guidelines [18].

As there was no clinical practice guideline for managing depression in the elderly in Norway, and only a national guideline on the management of depression among adults in general, we conducted a systematic review, assessing 13 national and international clinical practice guidelines for managing depression in primary care [19]. We identified all relevant recommendations for elderly patients with depression. We prioritised six of these recommendations for implementation.

### The six prioritised recommendations for managing depressed elderly patients

#### Social contact

Primary-care physicians and other health-care professionals should discuss social contact with elderly patients with depression, and recommend actions (for example, group activities) for those who have limited social contact. When needed, regular social contact should be provided with trained volunteers recruited from Centres for Voluntary Organisations, the Red Cross, Mental Health or community day care centres. When possible, the patient’s relatives should be involved in the plan to improve social contact.

#### Collaborative care

All municipalities should develop a plan for collaborative care for patients with moderate to severe depression. The plan should describe the responsibilities and communication between professionals who have contact with the patient, within primary care and between primary and specialist care. In addition, the plan should appoint depression care managers who have a responsibility for following the patient. The plan should describe routines for referral to specialist care. Municipalities are the atomic unit of local government in Norway and are responsible for outpatient health-care services, senior citizen services and other social services. There are 428 municipalities.

#### Depression care manager

Primary-care physicians should offer patients with moderate to severe depression regular contact with a depression care manager.

#### Counselling

Primary-care physicians or qualified health-care professionals should offer advice to elderly patients with depression regarding:

• Self-assisted programs, such as literature or web-based programs based on cognitive behavioural therapy (CBT)

• Structured physical activity programmes, individually or group based

• Healthy sleeping habits

• Strategies for coping with anxiety

• Problem-solving

#### Mild depression

Primary-care physicians should not prescribe antidepressants to patients with mild depression. Primary-care physicians may consider prescribing antidepressant medication to patients who suffer from a mild episode of depression and have previously responded to antidepressants when moderately or severely depressed.

#### Severe depression, recurrent depression, chronic depression and dysthymia

Primary-care physicians should offer these patients a combination of antidepressant medication and psychotherapy. If the physician is not trained to provide the patient with psychotherapy, patients should be referred to trained health-care professionals.

### Usual care

The following description of usual care for depression management in the elderly in Norway is based on clinical experience, government reports and scientific publications. We have also included international data, if data specific for Norwegian practice are lacking. We describe usual care with regard to the six prioritised recommendations.

#### Social contact

Isolation and loneliness are major risk factors for developing depression [20]. Although Norway has a scattered population, 44% live in one of the six largest city areas [21]. The proportion of elderly is higher in the rural areas. The recommendation addresses several levels of the municipality, from primary health-care providers (physicians, nurses and occupational therapists) and voluntary organisations. Some municipalities have included voluntary organisations and volunteers in their health-care planning, whereas others have no such collaboration. To some extent volunteers are involved in the follow-up of psychiatric patients of all ages. We believe that primary-care practitioners in general have neither routines nor procedures for involving volunteers in the management of elderly patients with depression.

#### Collaborative care

Although some municipalities and city districts have developed a general plan for managing patients with mental-health-related disorders, we believe that this is not the rule for most. A specific plan for depression care management is, at best, a part of such a plan. Specific plans for managing elderly patients with depression are absent. Many health-care regions have signed agreements on collaboration between the municipality and specialist health-care services. These statements are merely advisory, and do not dictate health-care providers’ behaviour.

#### Depression case manager

A Norwegian-register-based study found that approximately 1/3 of patients did not get a repeat prescription for antidepressants [22]. This may indicate that a more thorough follow-up, by a depression case manager, might improve the patients’ adherence to the treatment plan. The evidence for this service as part of a collaborative care plan is substantial [23,24], but has, to our knowledge, not been implemented or evaluated systematically in Norway. The service is intended to be an addition to the GP’s follow-up plan. Municipalities in Norway have developed community psychiatric nurse services to a large degree. These services do not serve all patients who need such care, and, when limited, we believe that elderly patients with depression are not prioritised in the community.

#### Counselling

This recommendation addresses several aspects of depression care management. The recommendation is addressed to health-care professionals. It is primarily relevant to GPs, but also to specially trained nurses. In addition, some sort of coordination with voluntary organisations or enterprises that offer physical training or activity programmes is needed. We believe that the actions in this recommendation are frequently used by practitioners, albeit not in a systematic or coordinated way. Evidence-based tools for providing advice on self-help, sleep problems, anxiety and problem-solving therapy will be identified and disseminated. Health-care professionals need to be trained for some of the actions, particularly problem-solving therapy and coping strategies. We believe that approximately 10% of GPs have some formal training in cognitive behavioural therapy, which also is useful.

#### Mild depression

This recommendation is addressed to GPs. We believe that current practice is characterised by prescribing antidepressants as soon as a diagnosis of depression is established, regardless of depression severity.

#### Severe depression, recurrent depression, chronic depression and dysthymia

This recommendation addresses primary-care professionals, primarily GPs and community psychiatric nurses, but also psychiatrists and psychologists in private practice and community psychiatric centres and geriatric psychiatric services, which are organised within the specialist health-care system. We believe that elderly patients with chronic or recurrent depression may be referred to and, to some extent, followed up by specialists, primarily in outpatient clinics of geriatric psychiatry and to a lesser extent in community psychiatric centres [16]. Elderly patients with these forms of depressive disorder are offered therapy by psychologists and psychiatrists in private practice to a very limited degree [22]. Most of these patients are offered pharmacotherapy, but they do not receive adequate psychotherapy.

### Tailored implementation

Tailored interventions are strategies that are designed to achieve changes in health-care practices based on an assessment of determinants of practice [25]. Determinants of practice are factors that can be barriers to or enablers of desired health-care practice. The factors that may affect practice are numerous, and can be found at all levels of the health-care system, including the organisational, professional and patient levels [26]. Within these levels, a variety of cognitive, emotional, economic and knowledge factors may affect the way health-care organisations and providers prioritise and provide services to patients and the way patients and their relatives adhere to the recommended care. Determinants of practice may vary across patient groups and settings. Thus, it is logical that determinants of practice should be identified in specific patient groups and health-care systems when recommendations for practice are implemented, and that the implementation interventions that are used should be tailored to address the identified determinants of practice. In the Tailored Implementation for Chronic Diseases (TICD) collaborative research project, we compared alternative methods for identifying determinants of practice and linking implementation strategies to identified determinants across countries and chronic diseases. The Norwegian component of the TICD project focuses on implementing recommendations for managing depressed elderly patients in primary care [27].

Although tailored interventions have been found to be effective, it is unclear how best to identify determinants of practice or how to tailor interventions to address the identified determinants [28]. In TICD, we developed a comprehensive checklist and worksheets to assist with the identification of determinants and tailoring of interventions [26]. The TICD checklist includes 57 items grouped in seven domains (guideline factors, individual health professional factors, patient factors, professional interactions, incentives and resources, capacity for organisational change and social, political and legal factors). We used the TICD checklist to identify key determinants of practice for each of the six prioritised recommendations for the management of depressed elderly patients. We used different methods to identify the determinants: brainstorming and structured focus groups with researchers, clinicians, nurses and patients; open and structured individual interviews with clinicians, nurses and patients, and a mailed survey of clinicians and nurses. Subsequently, we used different methods to identify and prioritise implementation strategies to address the key determinants that we had identified. These methods included independent assessments by the investigators informed by the TICD checklist, and unstructured and structured discussions in focus groups with clinicians, carers and other key stakeholders.

### Objectives

The objective of this study is to evaluate the effectiveness of tailored interventions to implement the six recommendations for the management of elderly patients with depression in primary care. The purpose of the trial is to inform decisions about how to improve care for depressed elderly patients in daily practice.

### Dissemination of results

The results of this study will be published in peer-reviewed journals and we will give oral and poster presentations at national and international conferences.

## Methods/design

### Trial design

We will conduct a pragmatic cluster randomised trial comparing the implementation of the six recommendations using tailored interventions with no intervention [29].

We will randomise 80 municipalities into one of two groups: an intervention group, to which we will deliver tailored interventions to implement the six recommendations, and a control group, to which we will not deliver any intervention. We will randomise municipalities rather than patients, individual clinicians or practices because we will deliver the intervention for the first three recommendations at the municipal level and we want to minimise the risk of contamination across practices for the other three recommendations.

**Figure 1:**
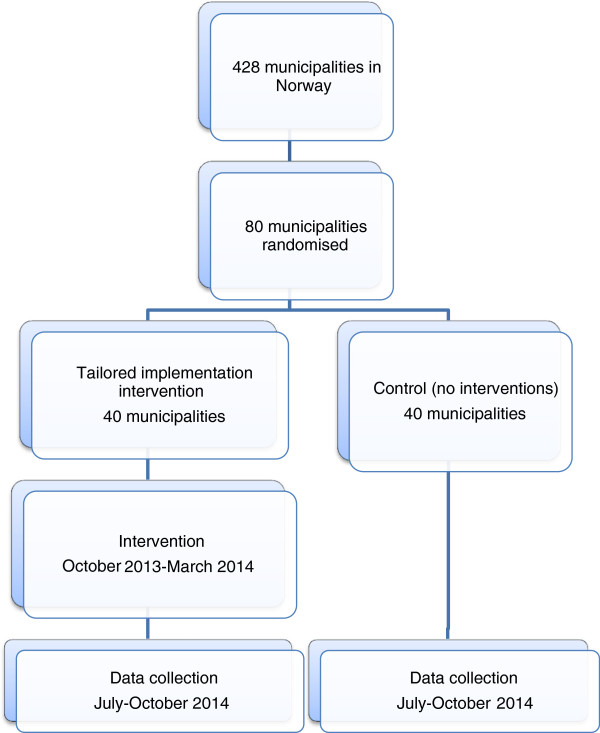
**Flowchart for the randomised controlled trial.** RCT, randomised controlled trial.

The flow of participants through the trial is shown in Figure 1. All 80 selected municipalities will be randomised at the start of the study. The tailored intervention will be delivered over six months, beginning in October 2013 and ending in March 2014.

### Participants and settings

We will include 80 municipalities. We have selected municipalities from these counties: Aust-Agder, Vest-Agder, Akershus, Oslo, Hedmark, Oppland and Troms (see Additional file 1: Appendix C for details of each municipality). The municipalities within these counties have been selected for pragmatic reasons (geographical access by the research team) and because they represent both urban and non-urban communities and large and small municipalities in Norway, and hence they are representative of the municipalities in Norway.

At the health-care professional level, we will include GPs in the data collection. Although the resources provided apply to and will be aimed at all health-care professionals in the municipalities (including specialists in private practice), we will mainly target our interventions at GPs because the GP practice is a core service for most elderly patients, and our primary outcome is measured at the GP level.

At the patient level, we will include elderly patients who live at home, 65 years or older, with a diagnosis of mild, moderate, severe or recurrent depression or assumed to have a diagnosis of depression according to standardised criteria (see Additional file 1: Appendix A) and who have consulted their practitioner within the last six months before the intervention.

### Eligibility criteria

All practising GPs in the included municipalities are eligible. Eligible patients will be identified by extracting information from GPs’ electronic medical records, using an algorithm based on the diagnostic codes in the International Classification of Primary Care, 2nd edition (ICPC-2). It is mandatory for Norwegian GPs to use this classification system in their contact with patients. Furthermore, the algorithm will contain ICPC2 diagnostic text, free text, prescription of antidepressants and billing codes. We will use several criteria to identify depressed elderly patients, even if they do not have a recorded diagnosis of depression, because many practitioners will use other diagnostic codes. The algorithm will give a score from one to six, the lower score indicating a lower probability that the patient is suffering from depression. A definite diagnosis and assessment of the severity of a patient’s depression will be based on the International Statistical Classification of Disease and Related Health Problems (ICD-10) [30] (in order to distinguish each case with regard to depression severity (mild, moderate or severe) and whether the patient suffers from recurrent or chronic depression or dysthymia. Patients will be excluded if they have a diagnosis of dementia, bipolar disorder or reside in nursing homes or are assessed by their practitioner to have low life expectancy.

From each GPs’ list we will choose a total of six patients with depression. Each list will be sorted using the score from the algorithm. We will choose patients from the top of the list. If a list contains more than six patients with a score of six, we will select from those patients randomly. If any of the first six patients identified do not suffer from depression according to the ICD-10 criteria, we will chose more patients from the list, until we have identified six patients with depression according to the ICD-10 criteria.

### ICD-10 classification of depression

These criteria are to be used in interviews with GPs to identify eligible patients and to grade the severity of depression.

The severity of a depressive episode is:

• mild (F32.0): at least two typical symptoms, plus at least two other common symptoms; none of the symptoms intense

• moderate (F32.1): at least two typical symptoms, plus at least three other common symptoms; some symptoms marked

• severe (F32.2): all three typical symptoms, plus at least four other common symptoms; some symptoms severe with intensity

• severe with psychotic symptoms (F32.3); as described in F32.2 but with delusions, psychomotor retardation or stupor so severe that ordinary social activities are impossible

A recurrent depressive disorder (F33) is where there are recurrent depressive episodes. Persistent mood disorders (F34.1) are known as dysthymia. An abridged depressive episode has a minimum duration of episode of about two weeks.

Typical symptoms (the core symptoms) are: depressed mood, loss of interest and enjoyment, reduced energy and increased fatigability.

Other common symptoms are: reduced concentration and attention, reduced self-esteem and self-confidence, ideas of guilt and unworthiness, agitation or retardation, ideas or acts of self-harm or suicide, disturbed sleep and diminished appetite.

### Implementation program

#### Development of the implementation program

In previous phases of the TICD project, we identified determinants for the implementation of the recommendations as well as strategies to address those determinants. This process is reported in detail elsewhere [25]. After prioritisation of the six recommendations, we discussed these in focus groups with health-care professionals, individual interviews with health-care professionals and patients, and we sent a survey to health-care professionals across the country. Numerous determinants of practice were identified. Using a standardised prioritisation method, which was also used by the other participating research groups in the TICD project, we prioritised 23 determinants. These determinants were discussed with implementation scientists, quality improvement professionals, stakeholder groups, general practitioners, nurses in primary care and relatives of elderly patients with depression, to suggest interventions that addressed the identified determinants. After prioritising and grouping the interventions that were suggested, we developed a comprehensive package of tailored interventions (the TICD package), in which each strategy addresses one or more specific determinants.

#### The logic model

The tailored interventions will consist of a package of strategies selected to address key determinants that we identified, which we assume affect the potential to improve the care of elderly patients with depression in primary care. A total of 52 strategies, addressing one or several determinants will be implemented. This package of strategies includes the following components:

1. Support for the development of a collaborative care plan by the municipality, including the management of elderly patients with moderate to severe depression.

a. Development of the plan: We will develop strategies that provide municipalities with tools and checklists for developing a collaborative care plan, including a list of the types of key personnel who should participate and in which form the recommendations should be presented to professionals in the municipality.

b. Content of the plan: We will provide a checklist of the content, including advice on the responsibility of health-care professionals, information on the availability of psychotherapy for moderately or severely depressed elderly patients, information about establishing services regarding depression care managers in the community, how to access voluntary services and a plan for dissemination and implementation of the plan. Resources for general practitioners and other health-care professionals. We will provide information on alternatives to antidepressants for mild depression, tools for counselling, tools for referral to depression care managers and psychotherapists and other tools and information that may help health-care professionals adhere to the recommendations.

2. Resources for patients and their relatives. We will provide information on alternatives to antidepressants for mild depression and evidence for counselling in depression and the combination of antidepressants and psychotherapy for severe depression, recurrent depression, chronic depression and dysthymia. Outreach visits to GP practices or GPs’ educational groups. We will discuss the recommendations with GPs, and adapt the content of the visits according to the needs of each practice. We will discuss how GPs prescribe antidepressants for mild depression and their feeling of having limited time. We will discuss the possibility for extended consultations and relevant fees for assessing the severity of depression and any other factors relevant for adherence to the recommendations.

3. Educational resources. We will provide information on training in cognitive behavioural therapy and counselling, and we will develop e-learning courses. We will provide resources for volunteers who need to improve their communication with depressed patients.

4. Data systems. We will develop a comprehensive website, with educational resources and tools for health-care professionals, patients and relatives.

**Figure 2:**
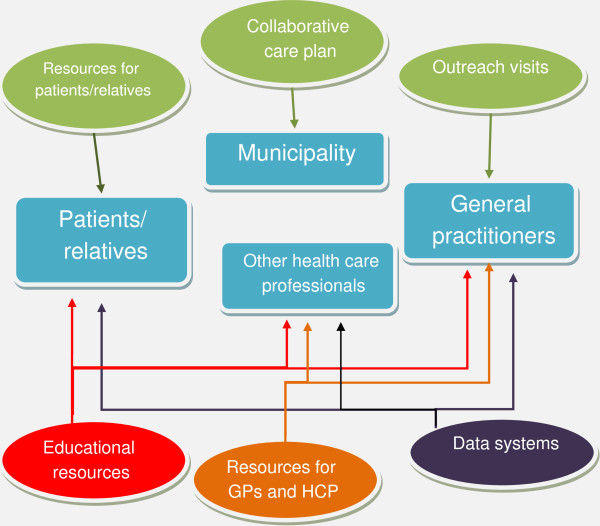
**How the interventions target various levels of the health-care system.** GP, general practitioner; HCP, health-care professional.

Figure 2 illustrates how the various components in the package target various levels of the health-care system.

For a detailed description of the strategies, and their relation to the determinants of practice and recommendation, see Additional file 1: Appendix B.

We have not addressed determinants and interventions that we considered unrealistic within this study. An example is the determinant ‘lack of health-care professionals to provide psychotherapy’ and the suggested intervention ‘employ more professionals who can provide psychotherapy’. Additionally, we omitted all suggested interventions that could compromise the randomised design of the trial, such as using the media (radio, newspapers and so on) and electronic resources available to all health-care professionals, such as the Norwegian Electronic Medical Handbook and the Norwegian Electronic Health Library.

### Outcome/measures

#### Primary outcome

The primary outcome is the proportion of recommendations that are implemented by the general practitioners. We will interview GPs to obtain this information for up to six included patients. The proportion of the prioritised recommendations adhered to for each patient will be measured. The questions for the GP will depend on the severity of the patient’s depression.

Mild depression (seven questions)

1. Did the GP offer the patient a self-help programme (web-based, book or course)?

2. Was the patient offered antidepressants? If yes, has the patient previously responded to antidepressants when moderately or severely depressed?

For questions 3 to 7, the GP will be asked if they discussed each of the following topics with the patient, and if the patient experienced the problem, were they offered advice about how to address the problem.^a^

3. Lack of social contact

4. Sleep problems

5. Lack of physical exercise

6. Anxiety

7. Difficulties problem-solving

Moderate depression (seven questions)

1. Did the GP offer the patient a self-help programme (web-based, book or course)?

2. Was the patient referred to a case manager?

For questions 3 to 7, the GPs will be asked if they discussed each of the following topics with the patient, and if the patient experienced the problem, were they offered advice about how to address the problem.^a^

3. Lack of social contact

4. Sleep problems

5. Lack of physical exercise

6. Anxiety

7. Difficulties problem-solving

Severe depression (two questions)

1. Was the patient referred to a case manager?

2. Were antidepressants prescribed and was the patient offered psychotherapy?

#### Secondary outcomes

We will measure the following patient outcomes for up to six patients for each GP. Most of these outcomes will be assessed by the patients themselves.

1. Global improvement in depression as assessed by the GP using the Clinical Global Impression Scale – Improvement (CGI-I) [31].

2. Improvement in depression as assessed by the patient or a family member:

• Patient Global Impression – Improvement (PGI) [32,33]. CGI-I and PGI are identical measures, using a 7-point Likert scale, where 1 means very much improved, and 7 is very much deteriorated.

• The presence of symptoms of depression and/or anxiety using the Hospital Anxiety and Depression Scale (HADS) [34]. HADS is a 14-item questionnaire, of which seven items measure depressive symptoms and seven items measure anxiety.

3. Loneliness [35,36]

• Do you sometimes experience loneliness? (0 = often, 1 = sometimes, 2 = seldom 3 = never)

4. Social contact

• Did you lack social contact when you first discussed being depressed with your GP? (yes/no)

• If yes, did you subsequently establish social contact with the help of a voluntary organisation or by other means? (0 = no, 1 = once only, 2 = more than once)

5. Physical activity

• Were you physically inactive when you first discussed being depressed with your GP? (yes/no)

• If yes, have you subsequently become more physically active? (yes/no)

6. Sleep problems

• Did you have a problem sleeping when you first discussed being depressed with your GP? (yes/no)

• If yes, has the sleeping problem improved?^b^

7. Anxiety

• Did you have a problem with anxiety when you first discussed being depressed with your GP? (yes/no)

• If yes, has your ability to cope with your anxiety improved?^b^

8. Problem-solving

• Did you have difficulties with problem-solving when you first discussed being depressed with your GP?

• If yes, has your ability to solve problems improved?^b^

9. Use of a self-help programme or reading self-help literature

• Have you used a self-help programme or read self-help literature? (yes/no)

10. Adherence with antidepressants

• Self-reported measure of medication adherence [37,38]

1. Do you ever forget to take your medication?

2. Are you careless at times about taking your medicine?

3. When you feel better, do you sometimes stop taking your medicine?

4. Sometimes when you feel worse, do you stop taking your medicine?

Each ‘yes’ scores 1, a score of 0 suggests no problem with medicine-taking and hence good compliance. The maximum of 4 for the four questions indicates major difficulties and suggests poor compliance.

We will assess whether the following items are present at the municipal level:

1. A collaborative care plan including a plan for elderly patients with depression (document)

2. An identifiable case manager

3. Agreed referral processes

4. Agreed communication processes within primary health-care services

5. Agreed communication processes between primary health-care and specialist health-care services

6. A list of voluntary organisations

7. Awareness of the collaborative care plan (proportion of GPs who are aware of the plan)

8. Knowledge of the collaborative care plan (proportion of GPs who can answer a factual question about the content of the plan)

We will collect data for the first six items above by questionnaires sent to municipality representatives. We will collect data for the last two items during practice visits at the end of the study.

### Sample size

We plan to include 80 municipalities. We estimate that there are an average of 3.68 practices per municipality and 2.97 GPs per practice (10.93 GPs per municipality), based on data from Statistics Norway and the Norwegian Medical Association. We conducted power calculations for the primary outcome (adherence to recommendations) assuming an alpha of 0.05 (risk of type I error), the ability to detect a minimum difference of 0.05 between the control group and the intervention group regarding GPs’ adherence to the recommendations (the primary outcome: the proportion of recommendations that are implemented by the general practitioners), a standard deviation of 0.17 and an intra-class correlation coefficient (ICC) of 0.02. We assumed that 40%, 50% or 60% of GPs will consent to data collection in samples with 60, 70 or 80 municipalities, respectively. Based on a previous study in which we randomised municipalities, we estimate that the ICC will be less than 0.03 [39]. Based on the assumption that most GPs currently adhere on average to 4 or less of the 7 or 8 recommendations per patient, and a pilot survey of 11 GPs, we estimate that the standard deviation most likely will be less than 0.2.

Based on these assumptions, our calculations indicate that the power to detect a minimum difference in adherence to the recommendations among GPs of 0.05 (scale 0 to 1) in a sample of 80 municipalities is 0.80 provided the standard deviation is 0.17, the ICC 0.02 and 50% of GPs consent to data collection. With 80 municipalities, 437 GPs should be included in the study and this would provide data for at most 2,622 patients. The detailed results of the power calculations are provided in Additional file 1: Appendix D.

### Recruitment

All 80 selected municipalities will be included in the study. We will seek consent from all GPs in the 80 municipalities, prior to data collection, after the intervention has been delivered. Participation in the intervention is optional; for example, GPs can elect to participate in an outreach visit or not. After the intervention, GPs in both the intervention and control groups will be invited to participate in the study as part of a free module-based course for continuing medical education on ‘Depression in the elderly’; however, for GPs in the control group this activity will start with data collection after the intervention. For this activity they will get credits for obtaining or renewing their speciality in general practice. During the course, we will collect data through an individual audit and feedback session. We will offer GPs who do not want to participate in the course the option of the audit and feedback session on its own. Patients will receive information about the study and a questionnaire. They will consent to participation by replying to the questionnaire and give informed consent.

#### Randomisation

A statistical consultant will randomise the municipalities. Computer-generated random numbers will be assigned to all 80 municipalities, without modifications in the group to which a municipality is randomly allocated. The municipalities will be divided into four strata based on information from Statistics Norway:

1. Municipalities with city status or a large population (>25,000 inhabitants)

2. Municipalities with a small population (≤25,000 inhabitants)

1. Municipalities with a high proportion of inhabitants aged 80 or older (>5%)

2. Municipalities with a low proportion of inhabitants aged 80 or older (≤5%)

From a representative selection of 80 municipalities from southern, eastern and northern Norway, we identified 19 municipalities with city status and/or a population larger than 25,000, 61 municipalities with a population ≤25,000, 46 municipalities with more than 5% of people over 80, and 34 municipalities with ≤5% of people 80 years or older (Additional file 1: Appendix C).

### Blinding

Blinding of the participants and the researchers regarding the intervention will not be possible. We will analyse and interpret the results without knowing the allocation. The interventions will be implemented before we contact the GPs to ask them to collect data for the study.

### Data collection

At the start of the study we collected the following baseline data for each municipality:

• Number of inhabitants, number of elderly patients (65+), the proportion of inhabitants aged over 80 and the number of GPs (from Statistics Norway and the municipalities)

• Whether the municipality has collaborative care plans for elderly patients with depression, for adult patients with depression, or for adults with mental health problems including for elderly patients with depression (collected by questionnaire with telephone follow-up if needed). If so, we will obtain a copy for further analysis

• Written agreements for routines for referral within primary care and between primary care and specialist health care

• A plan for depression care managers

• A plan for collaboration with voluntary organisations

• Whether the municipality is a member of the ‘Centre for Development of Institutional and Home Care Services’ network.

• Whether the primary-care-based psychiatric nurse teams provide regular services to the elderly population (65+)

We will collect outcome data beginning three months after the delivery of the intervention. In both groups we will collect data for all eligible patients using GPs’ medical records, structured interviews with GPs, brief questionnaires mailed to patients and questionnaires mailed to each municipality (with telephone follow-up, if needed). We anticipate that treatment strategies provided to patients with depression may take up to three months to prove beneficial.

Prior to contact with GPs, we will send them a program that will extract and identify eligible patients from their medical records. We will contact each GP practice that has consented to participate by telephone and identify eligible patients in their electronic medical records using the algorithm described in Additional file 1: Appendix A and collect the following information using structured telephone interviews with each GP:

• Depression severity according to ICD-10 for six patients identified with depression

• GP’s adherence to recommendations (primary outcome measure)

• GP’s assessment of a patient’s improvement, as measured CGI-I (secondary outcome measure)

• GP’s awareness and knowledge of a collaborative care plan in the municipality

If the GP faces any technical problems using the electronic device, we will provide support to solve the problem and if needed visit the GP to collect data.

Based on previous studies [40,41] we anticipate that between 40% and 60% of GPs will consent to data collection. All GPs that consent to participate will be asked to mail a questionnaire to each of the six patients that have been identified. Patients will be asked to complete and return the questionnaire by mail. If a patient consents to participate in the study, but does not wish to complete the questionnaire, we will ask the patient to appoint a family member to answer on their behalf if possible. Alternatively the patient may choose to consent to being contacted and interviewed by telephone.

The following data will be collected from patients:

• The patient’s assessment of improvement for depression, anxiety, sleep problems, physical activity, problem-solving, social contact and loneliness (secondary outcome measures)

• Adherence to medication

Each patient, GP and municipality included in the study will receive a unique study ID, which is available to the research group. A list at each GP practice will couple the patient’s study ID and the patient’s national ID number. We will use the patient ID numbers generated by the GPs’ electronic medical records systems. It will be possible for GPs to identify patients using these numbers, but the investigators will not have access to information identifying the patients, unless they first return a mailed consent form with the questionnaire. All communication (for example, letters) with patients will otherwise be through the patients’ GP.

We will collect the following descriptive data from GPs: age, sex, years of clinical experience as a GP, whether they are a specialist in primary-care medicine, competency in using CBT (collected during the structured interviews) and the number of elderly patients (65+) on the patient list.

A schematic presentation of participant timeline and time schedule for data collection is presented in Additional file 1: Appendix E.

### Statistical methods

The primary outcome of interest for our analysis is the mean adherence rate per GP (based on six patients). We will assess the number of recommendations that were followed with regard to the severity of depression, and we will calculate an overall mean for adherence across disease severity for each GP. For mild and moderate depression there are seven recommendations and for severe depression there are two recommendations; these will be assessed by interviewing the GP, who will have access to patients’ medical records during the interview. The analysis will be performed as an intention-to-treat analysis; we count all GPs in the group to which they were assigned, regardless of whether they received the intervention or not.

All analyses will be performed in SAS version 9.2 (SAS Institute Inc.) using PROC GLIMMIX with random effects for municipality and practice to account for the clustered nature of the data. Continuous data will be analysed assuming that the data follows a normal distribution (linear regression) and dichotomous data will analysed using the binomial distribution (logistic regression). In the initial analyses only the allocation to intervention or control will be included as an independent variable in the analysis (Intervention = YES/NO).

The following factors and variables are assumed to be effect modifiers: whether the municipality already has a collaborative plan (may improve adherence), whether access to cognitive behavioural therapy is poor (may reduce adherence), whether municipalities belong to the ‘Centre for Development of Institutional and Home Care Services’ network (may improve adherence) and whether GP has many elderly patients on the list (may improve adherence).

Each of the pre-specified effect modifiers will be separately included as independent variables (alongside allocation to intervention or control) in the model. All the effect modifiers with *P* < 0.3 in the previous step will be included as independent variables in a final multivariate model.

### Ethics

The intervention is a package of strategies targeted at municipal officials and health-care professionals with the aim of improving the delivery of recommended care to elderly patients with depression, and ultimately patient outcomes. Municipalities and GPs in the intervention group will be free to choose whether to use any of the intervention material that will be sent to them and GPs will be free to choose whether to participate in outreach visits or courses. Consequently, no consent is required prior to the intervention. Informed consent from GPs will be sought prior to data collection, which will take place after the intervention.

GPs will be given an identification number and all data collected from GPs will be stored, analysed and reported anonymously. The investigators will not collect information that enables them to identify individual patients unless a patient first gives written informed consent to a telephone interview, and all patient information will also be stored, analysed and reported anonymously. The participants may withdraw their consent at any time. This project has been approved by the Regional Ethical Committee of the South-Eastern region of Norway (file no 2013/572b).

## Discussion

This cluster randomised controlled trial will investigate whether a tailored implementation approach is an effective strategy for improving health-care professionals’ practice towards elderly patients with depression in primary care. The interventions target several prioritised determinants of practice. We will measure the overall effect of the implementation interventions as the proportion of GPs’ actions that are consistent with the recommendations. There is a risk of acquiescence bias when asking GPs whether they followed the recommendations. We intend to ask each GP about their treatment of the identified patients, and then check if the recommendations were followed or not. Thus, whether the GP is aware of the recommendations has less impact. The intervention is complex, and it will be difficult to identify the specific effect of each strategy in the intervention. The addressed determinants of practice were prioritised from hundreds of suggested determinants. Thus, there is a risk that, although we prioritised the determinants according to a common protocol for the TICD project, other determinants that we did not prioritise might, in fact, have a significant impact on practice. The planned process evaluation will explore these issues. The process evaluation will indicate whether we have identified the most important determinants and been able to develop and implement interventions that effectively address each of those determinants. To the extent that the interventions are not successful, the process evaluation will indicate if we have not identified or prioritised the most important determinants, or why the interventions did not adequately address the determinants that were identified.

## Trial status

The TICD project has been running since 2011, and will be concluded by the end of 2014. The intervention is planned to start autumn 2013.

## Endnotes

^a^For these recommendations, we will consider the recommendation implemented if the GP discussed the problem with the patient and the patient did not have the problem or if the patent had the problem and was offered advice

^b^We will use PGI to assess any improvement in sleep problems, anxiety and problem-solving.

## Abbreviations

CBT: Cognitive behavioural therapy; CGI-I: Clinical Global Impression Scale – improvement; GP: General practitioner; HADS: Hospital anxiety and depression scale; HCP: Health-care professional; ICC: Intra-class correlation coefficient; ICD-10: International statistical classification of disease and related health problems, 10th revised edition; ICPC-2: International classification of primary care, 2nd edition; PGI: Patient global impression – improvement; RCT: Randomised controlled trial; TICD: Tailored implementation for chronic diseases.

## Competing interests

The authors report no competing interests.

## Authors’ contributions

EA wrote the protocol. EA, ADO, SAF and IG planned the intervention. JO-J performed the statistical calculations. MW, ADO and SAF contributed to the protocol. All authors have approved the final version of the protocol.

## Supplementary Material

Additional file 1 **Appendix A.** Data extraction. **Appendix B.** Logic model. **Appendix C.** Municipalities and urban districts to be randomised. **Appendix D.** Power calculation. **Appendix E.** Participant timeline and time schedule for data collection. Click here for file
