# Supplementary material for: Tailored interventions to implement recommendations for elderly patients with depression in primary care: a study protocol for a pragmatic cluster randomised controlled trial
Source: Trials. 2014 Jan 9;15:16. doi: 10.1186/1745-6215-15-16 (PMC3899926; doi:10.1186/1745-6215-15-16)
Supplement: Additional file 1 — Appendix A. Data extraction. Appendix B. Logic model. Appendix C. Municipalities and urban districts to be randomised. Appendix D. Power calculation. Appendix E. Participant timeline and time schedule for data collection. [file 1745-6215-15-16-S1.docx]

# Additional file

# Captions

Caption, figure 2: Various interventions targeting various levels of the health care system. Green interventions target one level each. Other coloured interventions target several levels each.

# Appendix A Data extraction

1. Diagnose codes

International Classification of Primary Care – ICPC 2

| Symptoms and complaints   - P01 Feeling anxious/nervous/tense - P03 Feeling depressed - P27 Fear of mental disorder - P28 Limited function/disability - P29 Psychological symptom/complaint other | Other diagnoses   - P74 Anxiety disorder/anxiety state - P75 Somatisation disorder - P76 Depressive disorder - depression, - P77 Suicide/suicide attempt - P99 Psychological disorder, other |
| --- | --- |

1. Excerpts from additional diagnostic descriptions

Terms from ICPC-2 full text version (in Norwegian)

| Symptomer og plager   - P01 Følelse av angst/nervøsitet/anspenthet   - Angst, redsel - P03 Depresjonsfølelse   - Ulykkelig, utilstrekkelighet, bekymret - P05 Senilitet, atferd/følelse gammel   - Bekymring med aldring - P27 Engstelig for psykisk sykdom   - Bekymring for mental sykdom, frykt for å begå selvmord - P29 Psykiske symptomer/plager IKA   - Vrangforestillinger, multiple psykiske symptomer/plager | Other diagnoses   - P74 Angstlidelse   - angstnevrose - P75 Dissosiativ/somatoform lidelse   - Hypokondrisk lidelse - P76 Depressiv lidelse - P77 /selvmordsforsøk   - Suicidal atferd - P99 Psykisk lidelse IKA   - Nevrose IKA |
| --- | --- |

1. Excerpts from electronic medical journal

Norwegian terms from electronic medical journal that may resemble depression

| Deprimert, depres* (depresjon, depressiv, depresjonsskjema), deppa, nedfor, nedstemt* (nedstemt, nedstemthet) trist* (trist, tristhet, tristesse), sorgtung, svartsyn, håpløs* (håpløs, håpløst, håpløshet), tungsinn, mismot, ulykkelig, MADRS |
| --- |

1. Prescription of antidepressants

Prescription of antidepressants (ATC-codes, generic and brand labels available in Norway)

| N06A A04 Clomipramine (Anafranil, Klomipramin)  N06A A06 Trimipramine (Surmontil)  N06A A09 Amitriptyline (Sarotex, Sarotex retard)  N06A A10 Nortriptyline (Noritren)  N06A A12 Doxepin (Sinequan)  N06A B03 Fluoxetine (Fluoxetin, Fontex)  N06A B04 Citalopram (Cipramil, Citalopram)  N06A B05 Paroxetine (Paroxetin, Seroxat)  N06A B06 Sertraline (Sertralin, Zoloft)  N06A B08 Fluvoxamine (Fevarin) | N06A B10 Escitalopram (Cipralex, Esctitalopram)  N06A G02 Moclobemide (Aurorix, Moclobemid)  N06A X03 Mianserin (Mianserin, Tolvon)  N06A X11 Mirtazapine (Mirtazapin, Remeron)  N06A X12 Bupropion (Wellbutrin, Zyban)  N06A X16 Venlafaxine (Efexor, Venlafaxin, Venlix)  N06A X18 Reboxetine (Edronax)  N06A X21 Duloxetine (Cymbalta)  N06A X25 Hypericum (St John’s Wort) |
| --- | --- |

1. Consultation fees

| Fee for scoring with depression rating scale, extended consultation fees - 617, 615 |
| --- |

Extraction from these five sources within the patient’s journal will enable us to rate the probability for the patient having a depression. We will rate the patients according to the number of variables that apply to the patient, and rank the patients in the following order:

Score 6: An ICPC 2 diagnosis of depression (P76) within the last 2 years

Score 5: All variable categories apply to the patient (although diagnostic code P76 is not used) within the last two years

Score 4: Four variable categories used within the last two years apply to the patient and so on.

We will prior to the intervention pilot test the electronic medical journal data extraction software in two general practitioners’ patient journal system in order to check validity and feasibility. Software for data extraction will be elaborated in collaboration with Mediata/Medrave®

We will collect information in regard of repeat prescription.

Exclusion criteria: P70 Dementia P73 Affective disorder, bipolar disorder, mania and hypomania.

# Appendix B Logic model

**Logic model, part A: General principle of the logic model and overview**

Strategy/Intervention

Determinant

Recommendation

Improved implementation

Improved outcome (on patient, GP or municipality level

**Logic model, part B: The model**

1a. CCP-D Key personnel

2. CCP-D Model agreement

3. ER Educate volunteers

12. CCP-D Consider financial resources

11. CCP-D Create job description

10. RGP Contact information

4. RPR Inform relatives

1b. CCP-D Key personnel

5. CCP-D Help to obtain overview

6a. RPR Provide info to patients and relatives

7. OV Creative solutions

8. RPR Letters to patients

6b. RPR Info to patients on social contact, antidepressants

9. CCP-C Describe role of senior centres

A:1 Finding volunteers

A:2 Lack of awareness of local community /services

A:3 Social withdrawal in elderly patients with depression

A:4 Lack of connection between patient and volunteer

A:5 Requires organisation

A: Social contact

Will inform health care professionals and the community about the services in the community

A: Social contact

Will inform elderly and their relatives about the importance of social contact to alleviate depressive symptoms, and provide information regarding available resources

A: Social contact

Will provide information to establish the connection

A: Social contact

Will improve the chance that one suitable person will take the job of organising this

Assumed improved recruitment

Assumed improved awareness

Improved social contact as measured with loneliness scale and alleviation of depressive symptoms

Assumed improved connection between patient and volunteer

Assumed improved organisation

A: Social contact

Will help identifying volunteers, assist the municipality in establishing collaborative routines and help motivating people to volunteer for the task by improving skills to communicate with patients

B:1 Actionable plans with shared ownership increases the plan’s feasibility

B:2 Lack of coordination within municipalities, especially between GPs and other municipal services

B:3 Implementation of the plan

B: Collaborative Care Plan

Will include necessary key personnel to ensure sufficient adherence to the plan and provide tools and assistance to make the plan feasible.

B: Collaborative Care Plan

Will improve communication between health care professionals in the community

B: Collaborative Care Plan

Will help implementing the plan by providing guidance regarding recruitment and identification of eligible candidates for the case manager task, by ensuring that the plan is politically and administratively anchored and by providing advices regarding communication and using tools to implement the plan

Increased development of plans in the municipality, increased knowledge about the plan, measured in questionnaire to GPs

Increased referral to case manager as measured by GPs’ practice

Increased implementation of the plan in the municipality,

1c. CCP-D Include key personnel

23. CCP-C Monitoring and evaluation

24. CCP-D A modell plan with check list

25. DS Web page with all the resources and recommendations

26. CCP-D Arrangements for dissemination and implementation of the plan

22. CCP-C Help to implement the plan

21. CCP-D Politically and administratively anchored

20. CCP-C Clarify individual tasks

19. CCP-C Describe recruitment of CM

16b. CCP-D Dissemination and implementation plan

18. DS Support for e-communication

17. CCP-C Consistency with national plans

16a. CCP-D Help to develop a dissemination and implementation plan

15. CCP-D Exchange experiences

14. CCP-D Make it convenient to implement

13. CCP-D Include KS (The Norwegian Association of local & regional authorities

C:1 A description for how the GPshould proceed

27. OV Inform GPs about the concept and evidence supporting the CM

C: Case manager (CM)

Will improve GPs’ knowledge regarding the case manager and provide tools to approach case managers if needed

Increased referral to CM.

28a. RGP Structured referral forms to CM on web

29. CCP-C Establish CM services in each municipality

30a. CCP-C A plan for support/guidance for CM

31. ER Training for CMs in communication with depressed patients

32. ER Information CM regarding neccessity of family involvement

30b. CCP-C A plan for supervision groups for CMs, led by GPs, psychiatric nurses or specialist care

C:2 Good relationship between patient and CM

C:3 If the person is completely alone in the task

C: Case manager (CM)

Will provide strategies that improves communication and relationship between patients, their relatives and case manager

C: Case manager (CM)

Will reduce case managers feeling of professional loneliness

Assumed improved communication between patient and CM

Assumed reduced feeling of loneliness among CMs

33. OV Discuss time constraint and solutions

D:1 GPs’ time constraint

D: Counselling

Will inform GPs on the possibility to use extended consultation and additional fees for consultation, motivate GPs to offer counselling and look for alternatives if GP is not able to or don’t possess the skills to provide the service

Increased adherence to counselling

34. OV Clarify that elderly profit from counseling/psychotherapy

35. OV Consider other HCPs to offer psychotherapy

36. OV Inform GPs that this is effective

37a. RGP Brief information to discuss with patients

38. CCP-C Identify services in the community

37b. RGP Brief info on self help programmes etc

37c. RGP Check lists for counselling

39. ER Training in counselling as e-learning course

40. ER GP courses merits CME credits

41. ER E-learning and other courses to inform HCPs

D:2 Health professionals believe self-help program is not beneficiary for this population

D:3 There is a shortage of this type of service

D:4 GPs and health professionals’ lack of expertise regarding counselling

D: Counselling

Will inform health care professionals on the efficacy of non-pharmacological approaches in mild depression

D: Counselling

Will clarify whether this is a myth or not and offer tools for health care professionals to offer counselling

D: Counselling

Will help professionals to acquire the skills to provide counselling through courses, will provide tools to make counselling more feasible in clinical practice and motivate GPs to acquire the skills because courses are approved for speciality

Increased use of self-help programmes and exercise

Increased use of counsellingAssumed improved knowledge regarding services in the community

Increased adherence to counselling

33. OV Discuss time constraint and solutions

E:1 GPs’ time constraint

E: Mild depression

Will inform GPs on the possibility to use extended consultation and additional fees for consultation.

6c. RPR Patient info (brochures, web)

42. OV Provide evidence and alternatives

43. OV Emphasize the need for grading severity

44. OV Discuss the idea that GPs prescribe too rarely

45. ER Provide training in counselling

46. ER Courses merit for GPs’ speciality

47.ER E-learning and other courses

48. OV Discuss GPs urge to “do something” and that drugs are simple actions

E:2 Patient information that drugs do not help in mild depression

E:3 Difficult to reverse a trend where the doctor has been told that they prescribe antidepressants too rarely

E:4 Lack of other types of services makes it difficult to adhere

E: Mild depression

Will inform patients and their relatives that antidepressants in mild depression have limited or none expected clinical benefits but still they carry the risk of adverse effects.

E: Mild depression

Will inform GPs that non-pharmacological treatment strategies are effective, provide GPs with tools to target pharmacological treatment to patients with moderate and severe depression and provide an opportunity for the GP to express the feeling

E: Mild depression

Will improve the availability of health care professionals that possess the skills of counselling and motivate GPs to acquire the skills because courses are highly relevant and approved for speciality

Assumed less desire for ADs in mild depression

Increased adherence to counselling

Increased adherence to counselling

E:5 GP wants to "do something", drugs are simple actions

E: Mild depression

Will reduce the GP’s urge to “to something” (e.g. prescribe) by introducing alternatives to antidepressants

Reduced prescription of ADs in mild depression.

Reduced prescription of ADs in mild depression. Increased adherence to counselling

49. ER Training in cognitive therapy

F:1 GPs do not have this expertise (psychotherapy)

F: Severe depression, recurrent depression, chronic depression and dysthymia

Will increase the number of health care professionals that possess the skills of CBT and provide GPs with tools to refer

Increased number of patients with severe depression referred to psychotherapy Alleviation of depressive symptoms

28b. RGP Structured referral forms for psychotherapy

6d. RPR Information about combination therapy

1d. CCP-D Include key personnel

50. CCP-C A clear message in the plan about access to therapy

51. CCP-C A system of monitoring and evaluation of the plan

52. CCP-C State that recommendations are accordant with national plans

34. OV Clarify that elderly profit from counseling/psychotherapy

49. ER Training in cognitive therapy for GPs and nurses

F:2 Elderly are not prioritised for this type of service

F:3 Lack of health professionals who can provide this type of service

F: Severe depression, recurrent depression, chronic depression and dysthymia

Will emphasise that elderly should receive this service in the same degree as younger adults, by describing this in the plan and document that the recommendation is in accordance with national plans and by informing patients and their relatives so that this service may be requested in consultations

F: Severe depression, recurrent depression, chronic depression and dysthymia

Will increase the number of health care professionals that possess the skills of CBT, provide GPs with tools to refer and improve communication between GPs and specialists and health professionals that may provide psychotherapy.

Increased number of patients with severe depression referred to psychotherapy Alleviation of depressive symptoms

Increased number of patients with severe depression referred to psychotherapy Alleviation of depressive symptoms

**Logic modell, part C: Description of interventions**

This table comprises a comprehensive description of each intervention. The numbers refer to the numbers in the figures. Closely related strategies are given identical numbers, with ascending lower case letters.

| *1a. Collaborative care plan – development.* Include key personnel, e.g. leaders for voluntary organisations who can help identifying volunteers | *2. Collaborative care plan – development*. Provide a model agreement between the municipality and voluntary organisations that clarifies expectations, responsibilities (such as a contact or an office), communications (such as, for instance, a website, neighbourhood/local newspaper, "result"), follow-up and monitoring | *3. Educational resources.* Educate voluntaries in communication with depressed patients | *4. Resources for patients and their relatives.* Inform relatives, use existing local knowledge within the community (e.g. homebased nurse staff, voluntary organisations, *congregations*) | *1b. Collaborative care plan – development*. Include key personnel (e.g. families, GPs, home based nursing services, health centre for the elderly, municipality’s cultural agency, Council for the elderly and retired) |
| --- | --- | --- | --- | --- |
| *5. Collaborative care plan – development*. Help to obtain an overview of services in the community (collective overview in one place, e.g. by the home based nursing services administration, responsible for contacting voluntary organisations for an overview) | *6a. Resources for patients and their relatives.* Provide information e.g.via the council website, brochures and advertisements in the local newspaper | *7. Outreach visits.* Creative / alternative solutions for social contact (eg involving families, home care can identify depression) | *8. Resources for patients and their relatives.*  Outreach activities (e.g. letter to all over 80) | *6b. Resources for patients and their relatives.*  Information to patients and their relatives on social contact, alternatives to antidepressants and counselling (e.g. in brochures aimed at patients and their families, by contacting elderly who do not attend consultations or their relatives) |
| *9. Collaborative care plan – content.* Describe the role of senior centres and health clinics for the elderly in reducing social withdrawal | *10. Resources for general practitioners and other health care professionals.* Contact information for physical activity, voluntary organizations, senior centres, etc. (e.g. contact / coordinator of the municipal / district, using brochures) | *11. Collaborative care plan – development.* Create a job description that helps the municipality to find suitable persons who can lead the efforts | *12. Collaborative care plan – development.* Consider the financial resources to motivate people to take this work | *1c. Collaborative care plan – development.* Including key personnel in the development of the plan (e.g. coordinator / office for approval of health services, GP / GP committees, Community based psychiatric centres) impose key personnel to help in the development of the plan |
| *13. Collaborative care plan – development.* Include The Norwegian Association of Local and Regional Authorities (KS) and local opinion leaders in the work with the plan and presentation of recommendations | *14. Collaborative care plan – development.* Help to make it convenient to implement the plan (e.g., to create a comprehensive plan for psychiatry, where seniors also have a place | *15. Collaborative care plan – development.* Exchange experiences (good / bad) across municipalities | *16a. Collaborative care plan – development.* Help to develop a dissemination and implementation plan | *17. Collaborative care plan – content.* The plan must be consistent with the national collaboration reform |
| *18. Data systems.* Support for electronic communication between health care personnel in the community and specialists if possible | *16b. Collaborative care plan – development.* Help to develop a dissemination and implementation plan | *19. Collaborative care plan – content.* Describe the recruitment of care managers to obtain suitable personnel (use local knowledge to identify particularly suitable people) | *20. Collaborative care plan – content.* Clarify the individual tasks with clear guidelines and support for them to adhere, one person responsible for the plan (e.g. CMO) | *21. Collaborative care plan – development.* Include The Norwegian Association of Local and Regional Authorities (KS) and local opinion leaders in the work with the plan and presentation of the recommendations. The plan should be politically/ administratively anchored |
| *22. Collaborative care plan – content.* Help to implement the plan in practice, e.g. through regular meetings. If necessary to compel health professionals to implement the plan. | *23. Collaborative care plan – content.* Arrangements for monitoring and evaluation of the plan (e.g. via notification systems, involving health committee) | *24. Collaborative care plan – development.* A model plan with a checklist of both the process to make the plan and the content of the plan | *25. Data systems.* Web page with all the resources and recommendations | *26. Collaborative care plan – development.* Arrangements for dissemination and implementation of the plan |
| *27. Outreach visits to GPs.* Inform GPs about the concept and evidence supporting the CM, and how referral should be done | *28a. Resources for general practitioners and other health care professionals.* Structured referral forms to case manager, web-based | *29. Collaborative care plan* – content. Establish CM services in each municipality and effective referral practices of GPs to CM. Consider initiating contact between doctor, patient and CM. CM can be a GP assistant in the GP practice or another appropriate person in primary care. | *30a. Collaborative care plan* – content. A plan for support / guidance / counselling for CM | *31. Educational resources.* Training in communication with depressed patients for CMs |
| *32. Educational resources.* Inform CM that family members should be involved when necessary | *30b. Collaborative care plan* – content. A plan for support / guidance / counselling for CMs (e.g. establishing supervision groups for CMs led by GPs, psychiatric nurses or specialist care) | *33. Outreach visits to GPs*. Discuss physician time constraints and the possibility of extended consultations and additional fees | *34. Outreach visits to GPs.* Clarify to GPs that older with moderate to severe depression profit from counselling | *35. Outreach visits to GPs.* Consider if other health professionals than GPs can offer counselling |
| *36. Outreach visits to GPs.* Emphasize for GPs that we have alternatives to antidepressants for mild depression that are more effective and less harmful | *37a. Resources for general practitioners and other health care professionals.* Resources for counselling (e.g. brief information about self-help programs, physical activity, sleep habits and anxiety coping that can be discussed with patients and caregivers, use simple forms or manuals | *38. Collaborative care plan* – content. Identify available services for the patients in the municipality to determine if it is right that the services are missing | *37b. Resources for general practitioners and other health care professionals.* Resources for counselling: Brief info-material on self-help programs, physical activity, sleep habits and anxiety coping that can be discussed with the patient and their relatives/ caregivers | *37c. Resources for general practitioners and other health care professionals.* Resources for counselling: Simple forms / checklists |
| *39. Educational resources.* Training in counselling as PST, anxiety, coping and sleep habits, such as e-learning courses | *40. Educational resources.* Courses for GPs must merit for the speciality (CME credits) (15h) and can be a combination of web-based courses and educational meetings | *41. Educational resources.* E-learning courses and other forms of informing healthcare professionals about the recommendations and in particular techniques for counselling and motivation, training for GPs should be designed as a clinical topic course (CME credits) | *6c. Resources for patients and their relatives.* Information to patients and their relatives on social contact, alternatives to antidepressants and counselling (e.g. written info in brochures, websites | *42. Outreach visits to GPs.* Provide evidence for not using antidepressants for mild depression and inform that we have better alternatives |
| *43. Outreach visits to GPs.* Emphasize for GPs the need for grading the severity of depression using appropriate tools, such as MADRS, for diagnosis and follow-up | *44. Outreach visits to GPs.* Discuss the idea that GPs feel that they are accused of prescribing antidepressants too seldom | *45. Educational courses.* Provide training in counselling as problem solving therapy, anxiety coping and sleep habits, for instance as e-learning courses | *46. Educational courses.* Courses for GPs must merit for the speciality (15h) and can be a combination of web-based courses and meetings | *47. Educational courses.* E-learning courses and other courses to inform healthcare professionals about the recommendations and special techniques of counselling and motivation |
| *48. Outreach visits to GPs.* Discuss this with GPs. Suggest strategies to avoid prescribing antidepressants | *49. Educational courses.* Training in cognitive therapy for general practitioners and psychiatric nurses for those who want it | *28b. Resources for general practitioners and other health care professionals.* Structured referral forms to psychotherapy (to private specialists and Community based psychiatric centres and Old Age Psychiatry | *6d. Resources for patients and their relatives.* Information to patients and their families about the combined treatment (psychotherapy and antidepressants) | *1d. Collaborative care plan – development.* Include key personnel in the development of the plan (managers, administrators, specialists in private practices, GPs, GPs’ committees, nurses, specialist care, patients and relatives) |
| *50. Collaborative care plan – content.* A clear message in the plan about access to psychotherapy for the elderly with severe depression with community based psychiatric centres and private practitioners | *51. Collaborative care plan – content.* A system for monitoring and evaluation of the plan | *52. Collaborative care plan – content.* State that the recommendations are in accordance with national guidelines |  |  |

# Appendix C Municipalities and urban districts to be randomized

| **Municipalities or urban district** | Population | **No of practices** | No of GPs | >25 000 inhabitants or urban district | ≤ 25 000 inhabitants | > 5% 80 years or older | ≤5% 80 years or older |
| --- | --- | --- | --- | --- | --- | --- | --- |
| 0213 Ski | 28970 | 8 | 21 | x |  |  | x |
| 0214 Ås | 17284 | 8 | 12 |  | x |  | x |
| 0217 Oppegård | 25520 | 4 | 21 | x |  |  | x |
| 0228 Rælingen | 16170 | 3 | 11 |  | x |  | x |
| 0229 Enebakk | 10487 | 1 | 7 |  | x |  | x |
| 0233 Nittedal | 21454 | 4 | 14 |  | x |  | x |
| 0234 Gjerdrum | 6152 | 1 | 6 |  | x |  | x |
| 0220 Asker | 56447 | 13 | 39 | x |  |  | x |
| 0235 Ullensaker | 31044 | 6 | 28 | x |  |  | x |
| 0236 Nes i Akershus | 19462 | 4 | 12 |  | x |  | x |
| 0239 Hurdal | 2664 | 1 | 4 |  | x | x |  |
| 030101a Gamle Oslo | 44958 | 10 | 23 | x |  |  | X |
| 030109a Bjerke | 29090 | 6 | 18 | x |  |  | X |
| 030110a Grorud | 26777 | 5 | 18 | x |  |  | X |
| 030113a Østensjø | 47164 | 11 | 34 | x |  | x |  |
| 030114a Nordstrand | 47696 | 9 | 35 | x |  | x |  |
| 0402 Kongsvinger | 17522 | 7 | 14 | x |  |  | X |
| 0403 Hamar | 29045 | 9 | 32 | x |  | x |  |
| 0412 Ringsaker | 33191 | 7 | 26 | x |  |  | X |
| 0415 Løten | 7477 | 2 | 7 |  | x |  | X |
| 0417 Stange | 19190 | 6 | 18 |  | x | x |  |
| 0418 Nord-Odal | 5141 | 1 | 5 |  | x | x |  |
| 0419 Sør-Odal | 7859 | 3 | 5 |  | x | x |  |
| 0420 Eidsskog | 6288 | 1 | 5 |  | x | x |  |
| 0423 Grue | 5003 | 2 | 5 |  | x | x |  |
| 0425 Åsnes | 7606 | 4 | 8 |  | x | x |  |
| 0426 Våler (Hedm.) | 3844 | 1 | 3 |  | x | x |  |
| 0427 Elverum | 20152 | 6 | 18 |  | x | x |  |
| 0428 Trysil | 6752 | 1 | 6 |  | x | x |  |
| 0429 Åmot | 4336 | 1 | 6 |  | x | x |  |
| 0430 Stor-Elvdal | 2683 | 1 | 3 |  | x | x |  |
| 0501 Lillehammer | 26765 | 9 | 25 | x |  | x |  |
| 0502 Gjøvik | 29202 | 8 | 26 | x |  | x |  |
| 0519 Sør-Fron | 3193 | 5 | 5 |  | x | x |  |
| 0520 Ringebu | 4578 | 1 | 4 |  | x | x |  |
| 0513 Skjåk | 2300 | 1 | 3 |  | x | x |  |
| 0514 Lom | 2376 | 2 | 3 |  | x | x |  |
| 0515 Vågå | 3734 | 1 | 4 |  | x | x |  |
| 0517 Sel | 6005 | 1 | 5 |  | x | x |  |
| 0521 Øyer | 5095 | 4 | 5 |  | x | x |  |
| 0522 Gausdal | 6141 | 4 | 4 |  | x | x |  |
| 0516 Nord-Fron | 5827 | 1 | 7 |  | x | x |  |
| 0528 Østre Toten | 14747 | 5 | 13 |  | x | x |  |
| 0529 Vestre Toten | 12928 | 5 | 12 |  | x | x |  |
| 0532 Jevnaker | 6479 | 3 | 6 |  | x |  | X |
| 0533 Lunner | 8776 | 4 | 8 |  | x |  | X |
| 0534 Gran | 13439 | 5 | 11 |  | x | x |  |
| 0536 Søndre Land | 5761 | 1 | 6 |  | x | x |  |
| 0538 Nordre Land | 6768 | 2 | 5 |  | x | x |  |
| 0540 Sør-Aurdal | 3154 | 1 | 2 |  | x | x |  |
| 0541 Etnedal | 1408 | 1 | 2 |  | x | x |  |
| 0542 Nord-Aurdal | 6428 | 1 | 9 |  | x | x |  |
| 0543 Vestre Slidre | 2239 | 1 | 3 |  | x | x |  |
| 0544 Øystre Slidre | 2232 | 1 | 3 |  | x | x |  |
| 0545 Vang | 1617 | 1 | 3 |  | x | x |  |
| 0904 Grimstad | 21301 | 5 | 19 | x |  |  | X |
| 0906 Arendal | 42801 | 15 | 37 | x |  |  | x |
| 0912 Vegårshei | 1933 | 2 | 2 |  | x | x |  |
| 0914 Tvedestrand | 6019 | 2 | 6 |  | x | x |  |
| 0919 Froland | 5257 | 2 | 5 |  | x |  | x |
| 0926 Lillesand | 9878 | 3 | 7 | x |  |  | x |
| 0928 Birkenes | 4828 | 1 | 5 |  | x |  | x |
| 0929 Åmli | 1818 | 1 | 2 |  | x | x |  |
| 1002 Mandal | 15149 | 3 | 13 | x |  |  | x |
| 1014 Vennesla | 13583 | 1 | 13 |  | x |  | x |
| 1017 Songdalen | 6165 | 2 | 6 |  | x |  | x |
| 1018 Søgne | 10855 | 1 | 11 |  | x |  | x |
| 1021 Marnardal | 2286 | 1 | 3 |  | x | x |  |
| 1027 Audnedal | 1689 | 2 | 2 |  | x |  | x |
| 1029 Lindesnes | 4753 | 2 | 5 |  | x |  | x |
| Kristiansand | 83246 | 22 | 74 | x |  | x |  |
| 1920 Lavangen | 1016 | 1 | 2 |  | x |  | x |
| 1923 Salangen | 2214 | 1 | 3 |  | x | x |  |
| 1925 Sørreisa | 1925 | 3 | 4 |  | x | x |  |
| 1933 Balsfjord | 5502 | 2 | 6 |  | x |  | x |
| 1936 Karlsøy | 2355 | 1 | 3 |  | x |  | x |
| 1938 Lenvik | 11455 | 7 | 16 |  | x | x |  |
| 1938 Lyngen | 3028 | 2 | 4 |  | x |  | x |
| 1939 Storfjord | 1909 | 1 | 2 |  | x | x |  |
| 1942 Nordreisa | 4807 | 1 | 6 |  | x |  | x |
| **Sample** | 1054392 | 298 | 894 | 19 | 61 | 46 | 34 |

# Appendix D Power calculation

| Number of municipalities | | 60 | 60 | 60 |  | 60 | 60 | 60 |
| --- | --- | --- | --- | --- | --- | --- | --- | --- |
| ICC |  | 0,02 | 0,02 | 0,02 |  | 0,02 | 0,02 | 0,02 |
| Proportion of GPs participating | | 0,4 | 0,5 | 0,6 |  | 0,4 | 0,5 | 0,6 |
| Minimal detectable difference | | 0,05 | 0,05 | 0,05 |  | 0,1 | 0,1 | 0,1 |
|  |  |  |  |  |  |  |  |  |
|  |  |  |  |  |  |  |  |  |
| GPs in sample | | 262,3104 | 327,888 | 393,4656 |  | 262,3104 | 327,888 | 393,4656 |
| Design effect | | 1,198592 | 1,198592 | 1,198592 |  | 1,198592 | 1,198592 | 1,198592 |
|  |  |  |  |  |  |  |  |  |
| Efective sample size | | 218,8488 | 273,561 | 328,2732 |  | 218,8488 | 273,561 | 328,2732 |
|  |  |  |  |  |  |  |  |  |
| STD |  |  |  |  |  |  |  |  |
| **0,17** |  | **0,58** | **0,679** | **0,757** |  | **0,991** | **0,998** | **>0.99** |
|  |  |  |  |  |  |  |  |  |
| Number of municipalities | | 70 | 70 | 70 |  | 70 | 70 | 70 |
| ICC |  | 0,02 | 0,02 | 0,02 |  | 0,02 | 0,02 | 0,02 |
| Proportion of GPs participating | | 0,4 | 0,5 | 0,6 |  | 0,4 | 0,5 | 0,6 |
| Minimal detectable difference | | 0,05 | 0,05 | 0,05 |  | 0,1 | 0,1 | 0,1 |
|  |  |  |  |  |  |  |  |  |
|  |  |  |  |  |  |  |  |  |
| GPs in sample | | 306,0288 | 382,536 | 459,0432 |  | 306,0288 | 382,536 | 459,0432 |
| Design effect | | 1,198592 | 1,198592 | 1,198592 |  | 1,198592 | 1,198592 | 1,198592 |
|  |  |  |  |  |  |  |  |  |
| Efective sample size | | 255,3236 | 319,1545 | 382,9854 |  | 255,3236 | 319,1545 | 382,9854 |
|  |  |  |  |  |  |  |  |  |
| Power at various std's | |  |  |  |  |  |  |  |
| **0,17** |  | **0,646** | **0,746** | **0,818** |  | **0,997** | **>0.99** | **>0.99** |
|  |  |  |  |  |  |  |  |  |
|  |  |  |  |  |  |  |  |  |
|  |  |  |  |  |  |  |  |  |
| Number of municipalities | | 80 | 80 | 80 |  |  |  |  |
| ICC |  | 0,02 | 0,02 | 0,02 |  |  |  |  |
| Proportion of GPs participating | | 0,4 | 0,5 | 0,6 |  |  |  |  |
| Minimal detectable difference | | 0,05 | 0,05 | 0,05 |  |  |  |  |
|  |  |  |  |  |  |  |  |  |
|  |  |  |  |  |  |  |  |  |
| GPs in sample | | 349,7472 | 437,184 | 524,6208 |  |  |  |  |
| Design effect | | 1,198592 | 1,198592 | 1,198592 |  |  |  |  |
|  |  |  |  |  |  |  |  |  |
| Efective sample size | | 291,7984 | 364,748 | 437,6976 |  |  |  |  |
|  |  |  |  |  |  |  |  |  |
| Power at various std's | |  |  |  |  |  |  |  |
| **0,17** |  | **0,707** | **0,799** | **0,867** |  |  |  |  |

# Appendix E Participant timeline and time schedule for data collection

Data collection (DC) and practice visits (PV)

| Year | 2013 | | | | | | 2014 | | | | | | | | | | | |
| --- | --- | --- | --- | --- | --- | --- | --- | --- | --- | --- | --- | --- | --- | --- | --- | --- | --- | --- |
| Month | Apr-Aug | Sep | Intervention: Oct 13 to Mar 14 | | | | | | Apr | May | | Jun | | Jul | | Aug | | Sep |
| Month No | ÷6 - ÷2 | ÷1 | Month 1 – 6 | | | | | | 7 | 8 | | 9 | | 10 | | 11 | | 12 |
| Intervention  (6 months) |  |  |  | | | | | |  |  | |  | |  | |  | |  |
| **Activity** |  |  |  |  |  |  |  |  |  |  | |  | |  | |  | |  |
| Planning the intervention | × | × |  |  |  |  |  |  |  |  | |  | |  | |  | |  |
| Practice  visit |  |  | × | × | × | × |  |  |  |  | |  | |  | |  | |  |
| Identifying eligible patients |  |  |  |  |  |  |  |  |  | | | | | ×  Both groups: Patients in contact with GP month ÷6 - 6 | | | | |
| **DC** |  |  |  |  |  |  |  |  |  | |  | |  |  |  | |  | |
| DC  Municipality | ×  Municipality  Baseline characteristics |  |  |  |  |  |  |  |  | |  | |  |  |  | | × | |
| DC GPs control |  |  |  |  |  |  |  |  |  | |  | |  | X | X | | X | |
| DC GPs intervention |  |  |  |  |  |  |  |  |  | |  | |  | X | X | | X | |
| DC patients control |  |  |  |  |  |  |  |  |  | |  | |  | X | X | | X | |
| DC patients intervention |  |  |  |  |  |  |  |  |  | |  | |  | X | X | | X | |
